# An *in silico *analysis of T-box regulated genes and T-box evolution in prokaryotes, with emphasis on prediction of substrate specificity of transporters

**DOI:** 10.1186/1471-2164-9-330

**Published:** 2008-07-14

**Authors:** Michiel Wels, Tom Groot Kormelink, Michiel Kleerebezem, Roland J Siezen, Christof Francke

**Affiliations:** 1 TI Food and Nutrition, Wageningen, The Netherlands; 2 CMBI, Radboud University Nijmegen-Medical Centre/NCMLS, Nijmegen, The Netherlands; 3 NIZO food research, Ede, The Netherlands

## Abstract

**Background:**

T-box anti-termination is an elegant and sensitive mechanism by which many bacteria maintain constant levels of amino acid-charged tRNAs. The amino acid specificity of the regulatory element is related to a so-called specifier codon and can in principle be used to guide the functional annotation of the genes controlled via the T-box anti-termination mechanism.

**Results:**

Hidden Markov Models were defined to search the T-box regulatory element and were applied to all completed prokaryotic genomes. The vast majority of the genes found downstream of the retrieved elements encoded functionalities related to transport and synthesis of amino acids and the charging of tRNA. This is completely in line with findings reported in literature and with the proposed biological role of the regulatory element. For several species, the functional annotation of a large number of genes encoding proteins involved in amino acid transport could be improved significantly on basis of the amino acid specificity of the identified T-boxes. In addition, these annotations could be extrapolated to a larger number of orthologous systems in other species. Analysis of T-box distribution confirmed that the element is restricted predominantly to species of the phylum Firmicutes. Furthermore, it appeared that the distribution was highly species specific and that in the case of amino acid transport some boxes seemed to "pop-up" only recently.

**Conclusion:**

We have demonstrated that the identification of the molecular specificity of a regulatory element can be of great help in solving notoriously difficult annotation issues, e.g. by defining the substrate specificity of genes encoding amino acid transporters on basis of the amino acid specificity of the regulatory T-box. Furthermore, our analysis of the species-dependency of the occurrence of specific T-boxes indicated that these regulatory elements propagate in a semi-independent way from the genes that they control.

## Background

Transcription anti-termination is a regulatory mechanism commonly encountered in all lineages within the bacterial kingdom (see e.g. [1-3]). In transcription anti-termination, the regulation of transcription occurs after the initiation of RNA synthesis, but before transcription of the coding region. The mechanism of anti-termination involves a structural change in the RNA transcript that is dependent on the interaction of the transcript with, for instance, a regulatory protein [4], a tRNA [5] or a metabolite [6]. The structural elements that compose these anti-terminators are encoded by conserved sequences on the DNA and can be found by searches for the related sequence motifs in upstream regions of regulated genes [7].

A well-studied anti-termination element is the so-called T-box. T-box anti-termination is an elegant and sensitive mechanism by which many bacteria maintain constant levels of tRNA charged with amino acids [2,8]. When there is a sufficient supply of charged tRNA in a cell, the T-box folds into a terminator structure, thereby blocking further transcription. Transcription can only proceed upon conversion into an anti-terminator structure, which is induced by binding of a highly conserved 5'-NCCA-3' of the uncharged tRNA with a conserved '5-UGGN-3' sequence in the T-box [9-11]. Although anti-terminator formation involves contacts between many nucleotides, the specificity of the interaction seems largely dependent on the interaction of a tri-nucleotide (anti-anti)-codon in the so-called specifier loop of the T-box with the anti-codon of an amino acid-specific tRNA [12-15]. The structural and kinetic details of this interaction have been well-studied [16-22]. The appropriate assignment of the specifier codon has been used previously to improve the functional annotation of various genes located downstream of the T-box [3,23-31]. The T-box controlled genes identified thus far encode functionalities that reflect perfectly the pivotal role of uncharged tRNAs in the regulatory mechanism. These functionalities include not only tRNA ligation, but also amino acid biosynthesis and transport [11,17,24,26,28,32-34]. The encoded proteins are involved in modulation of the level of uncharged tRNA in the cell, either directly by charging the corresponding tRNA with its cognate amino acid or indirectly by controlling the intracellular concentration of the specific amino acid.

To date, T-boxes have been identified predominantly in the genomes of bacterial species of the phyla *Firmicutes *(including *Mollicutes*) and *Actinobacteria *[7], although anti-termination systems have been argued to be among the oldest regulatory systems in bacteria because of their independence of regulatory proteins [35]. To investigate this further, we have explored the occurrence of T-boxes in all sequenced prokaryotic genomes. To circumvent potential differences between T-box systems in different bacterial lineages, an iterative HMM-based identification search was performed using the best conserved region of the T-box sequence. Species- and amino acid-specific T-box regulation networks were reconstructed. Most importantly, the acquired knowledge on amino acid specificity could be used to propose an improved functional annotation for many T-box controlled genes and to shed light on the evolution of the regulatory element itself.

## Results and discussion

### I) A comprehensive collection of T-boxes

**Figure 1:**
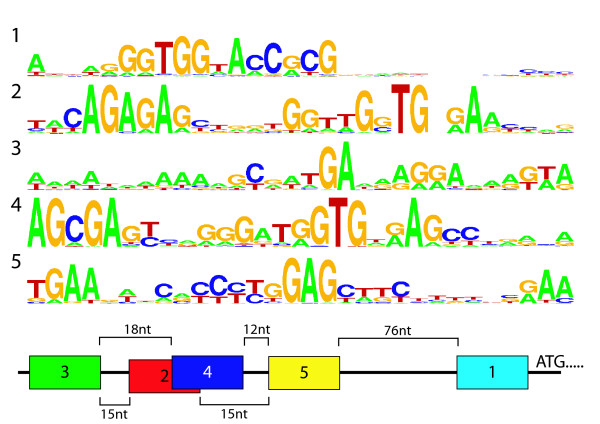
**Sequence logo**[64]**visualization of the 5 different T-box motifs.** Both the consensus sequence and relative conservation of individual residues is displayed. Motif 1 (information content: 19.2 bits) displays the motif used to perform the T-box identification. Validation was performed by checking the presence of motif 2 (29.7 bits) or 3 (20.9 bits) together with motifs 4 (31.4 bits) and 5 (25.8 bits). Motif 1 includes the (a-specific) tRNA interaction site (T-box sequence, consensus GGTGG) located in the antiterminator loop. The other motifs include different parts of the specifier loop [23,26]: GNTG- and AG-box in motif 2 and 4, TGA-, AGGA- and AGTA-box in motif 3 and a conserved part of the specifier loop in motif 5 (GAG). The specifier codon is to be found within 1 – 5 nucleotides upstream of this conserved GAG.

The analysis of the taxonomic and functional distribution of T-boxes was started by *de novo *identification of T-box motif characteristics. Conserved nucleotide sequence motifs upstream of tRNA-ligase encoding genes in species of the phylum *Firmicutes *were recovered and used to identify T-boxes located at other positions in the same genome as well as in the genomes of other species (see methods). These searches showed that a T-box could be specified best by a 30 nt motif that is extremely well-conserved and positioned in the 3'-region of the terminator/anti-terminator loop (motif 1 in Figure 1). In fact, this motif is known as 'the T-box sequence' since its discovery [25]. Later it was recognized that this conserved region belongs to a larger conserved RNA structure known as the T-box element [9]. This element contains four other highly conserved regions (see Figure 1 motifs 2–5).

The initial search showed prominent variations in the number of T-boxes per genome between different classes of the phylum *Firmicutes *and between different phyla. Therefore, additional searches with phylum-specific and class-specific T-box HMMs were performed, but generally did not yield novel hits. Only in the case of the *Clostridia *a limited number of 10 additional T-boxes were identified. Further iterations did not expand the dataset. Visual inspection of the upstream regions of all genes encoding a t-RNA ligase in the Firmicutes indicated that indeed all those regions that contain the distinctive T-box motifs were identified by our algorithm. A comparison of the number of T-boxes identified by us for a representative set of organisms with the number obtained using the Rfam T-box model [36] proved that our recovery procedure was very efficient (see methods for details).

#### Identification of the specifier codon and amino acid specificity

Although T-boxes were readily identified, it was more difficult to define their amino acid specificity. To that end, the phylogeny of homologous genes preceded by a T-box from different species was determined and the upstream regions corresponding to each orthologous group were aligned. In all the cases of T-boxes for which the specifier codon had been identified experimentally [3,5,9,11,12,14-18,21,28,29,33], we observed that the specifier codon aligned perfectly within the related orthologous sequences. In fact, this was true for almost all orthologous groups of sequences. Moreover, most of the alignments could easily be clustered by eye into larger groups for which the specifier codon remained directly apparent from the alignment. The resulting alignments and the annotation of the specifier codon can be found at [37]. Nevertheless, there remained a few less clear cases. For some (~5%) a secondary structure prediction could be used to provide the additional information required to define the specifier codon in the specifier loop [38]. Taken together, a specifier codon could be identified directly for over 90% of the identified T-boxes.

#### Codon usage in the specifier codon

Most amino acids are encoded by multiple codons. Leu for instance, is encoded by six different codons (CUA, CUU, CUG, CUC, UUA and UUG). Remarkably, the T-boxes had a conserved preference for certain codons within as well as between species (Additional file 1). Evaluation of these preferences showed that they complied almost perfectly with the rules observed by Elf et al. for the codon usage by *E. coli *[39]. In an elegant study these authors analyzed the dependence of the charging of various codon-specific tRNAs on the use of various codons in particular proteins. They concluded that: "when codon reading is part of a control loop that regulates synthesis of missing amino acid, the translation rate of the selected codon should be as sensitive as possible to starvation" [39]. And, in their paper they showed which codons are the most sensitive in *E. coli*. We found that for all but one of the most predominantly used specifier codons in T-boxes, the corresponding tRNA is among the highest in sensing shortage of that specific amino acid in *E. coli *as reported by [39]. The only exception was the T-box codon for Ala (GCU). Therefore, assuming the conclusions by Elf et al. are also valid for Gram-positive bacteria, our findings suggest that the codons that are sensitive to depletion are preferentially used in T-box regulation.

#### Functionalities controlled by a T-box

As expected, the proposed regulatory role of the T-box elements appeared to be perfectly reflected by the genes under their control. The majority of the T-boxes (62%) were found to precede genes encoding tRNA ligases, while most others were found upstream of genes encoding proteins involved in amino acid transport (12%) or amino acid biosynthesis (18%). The remaining T-boxes (8%; 71 genes in total) were found upstream of genes encoding proteins with unknown function (54 genes), or a function that lacks an apparent relation to amino acid metabolism (17 genes). A complete and species-specific subdivision of T-boxes based on function prediction of the proteins encoded downstream and a list of genes with no apparent relation to amino acid metabolism is provided in the supplementary material, in Additional files 1 and 2.

### II) The use of regulator specificity to improve annotation of molecular function and biological role

We made two important observations: i) In all cases, the T-boxes identified upstream of the genes encoding a tRNA ligase contained a specifier codon that corresponded with the amino acid specificity of the ligase; and ii) in all other cases where the function of the protein encoded by the gene downstream of the T-box had experimentally been verified, the specifier-codon corresponded to the established functionality of the gene. These observations implied that the employed method for the identification of T-box specificity was reliable and, consequently, that predicted T-box specificities could be extrapolated to the molecular function of the protein encoded by the gene located downstream, as had occasionally been done before. Many of the genes preceded by a T-box had not been specifically annotated to date in the sense that, although the functional category was often evident (e.g. proton symport, ABC transport family, etc.), a specific molecular function had not been attributed. In fact, more than two-third of the non-tRNA ligase genes preceded by a T-box lacked such a specific annotation of molecular function. As importantly, the functional annotation of the genes could be extended to a different level entirely by using the knowledge on T-box (regulator) specificity, as this knowledge discloses (in part) under which conditions the regulated genes will play their biological role (for the distinction between molecular function and biological role see Francke et al. [40]).

#### a) T-box regulation of amino acid transport

Many of the genes encoding amino acid transporters were found to be preceded by a T-box, especially in the genomes of the *Lactobacilli *and *Bacilli *of the *Bacillus cereus*-group. The transporters controlled by T-boxes belonged to no less than seven distinct transporter families (MFS, 2.A.1; APC, 2.A.3; NSS, 2.A.22; DAACS, 2.A.23; LIVCS, 2.A.26; NhaC, 2.A.35; and ABC-cassette, 3.A.1; Transporter Classification described by Saier [41]). Table 1 gives an overview of the distribution of the transport systems regulated by a T-box over the various Firmicutes species.

**Table 1:** Overview of T-box regulated transporter genes in different *Firmicutes*. The type and number (between brackets) of transporters are displayed per species and according to their predicted specificity.

	*B. anthracis *Ames 0581	*B. licheniformis *ATCC 14580	*B. subtilis *168	*O. iheyensis *HTE813	*L. monocytogenes *EGD-e	*L. plantarum *WCFS1	*L. acidophilus *NCFM	*L. johnsonii *NCC 533	*E. faecalis *V583	*L. Lactis *IL1403	*S. pneumoniae *R6	*C. acetobutylicum *ATCC824	*C. perfringens *ATCC13124	*C. tetani *E88
Asn							ABC							
Asp								ABC						
His					ABC	ABC	ABC		ABC					
Ile							ABC	ABC				ABC		
	LIVCS					LIVCS	LIVCS	LIVCS				LIVCS	LIVCS	LIVCS
Leu		APC	APC											
														LIVCS
	NSS													
Lys	MFS													
Met						ABC (5)	ABC (3)	ABC	ABC (3)	ABC				
Phe	NSS													
Thr	APC													
	LIVCS													
Trp						ABC					ABC			ABC
	NSS													
Tyr	NHAC			NHAC		NHAC (2)	NHAC	NHAC	NHAC					
														NSS
Val						LIVCS	LIVCS							
?						ABC							DAACS	

ABC					1|73	8|77	6|48	3|59	4|79	1|57	1|78	1|93		1|60
APC	1|19	1|20	1|18											
LIVCS	2|6					2|3	2|3	2|2				1|1	1|3	2|4
MFS	1|69													
NHAC	1|4			1|3		2|2	1|1	1|1	1|2					
NSS	3|4													1|5

At the bottom the total fraction of regulated transporters is shown per family. ABC: ATP-binding cassette superfamily, APC: Amino acid-polyamine-organocation family, DAACS: dicaboxylate/amino acid:cation symporter family, LIVCS: leucine/isoleucine/valine cation symporter family, MFS: major facilitator superfamily, NHAC: Na^+^:H^+ ^antiporter family, NSS: neurotransmitter:sodium family. Classification adopted from Saier [41].

Overall, for more than 85% of the T-box regulated transporters the functional annotation (molecular function and/or biological role) could be improved as compared to the entries in the reference database of NCBI. A full list can be found in Additional file 1. We have limited the substrate specificity definition in our annotation to putatively dominant substrates based on the amino acid specificity of the T-box. However, broader substrate specificity is probably more common for transporters. Especially transport systems consisting of only a permease are expected (and have been shown) to display broader substrate specificity (see [42] and [43] for examples), whereas systems that require prior substrate-binding (like in ABC transport) will be more specific. We discuss the T-box based functional annotation of some transport systems in more detail in the following paragraphs and in Additional file 3.

#### The ABC family

**Figure 2:**
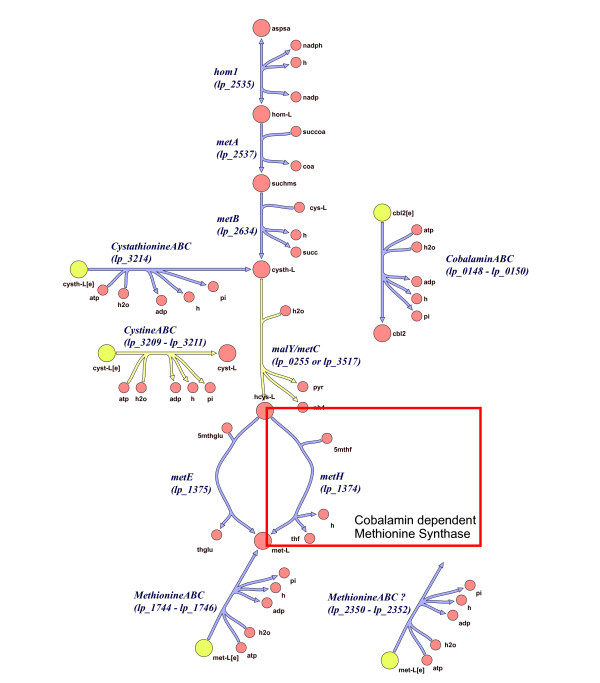
**Overview of T-box-regulated methionine biosynthesis in *L. plantarum*.** Reactions coloured in blue are catalyzed by proteins encoded by genes regulated by a T-box. The figure was generared using the the Simpheny software tool (Genomatica, San Diego, USA). Reactions were based on the metabolic model of *L plantarum *published by [65].

T-box regulation of ABC transport systems was found in most lineages of the *Firmicutes *but not in the *Bacilli*. The T-box regulated ABC transporters could be sub-divided into four sub-families, based on the specificity of the substrate-binding protein and the permease. A striking use of extensive T-box regulation in ABC transport was observed in *L. plantarum*. It appears that in the absence of methionine, *L. plantarum *uses a single mechanism to switch on not only transport of the amino acid itself, but also of the precursors and co-factors needed for its biosynthesis (see Figure 2 and Additional file 3).

#### The APC family

**Figure 3:**
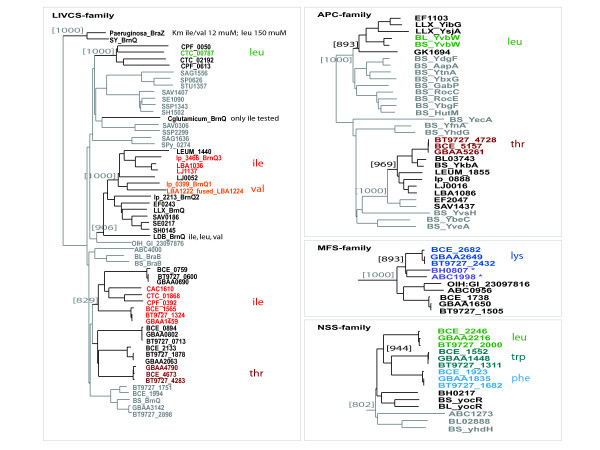
**Occurrence of T-boxes in relation with different transporter families.** The figure displays a NJ-tree of the LIVCS-family transporters of the Firmicutes (left) and partial trees related to the APC-, MFS- and NSS-family transporters. It appears T-boxes are only associated with very few proteins of these families and the association appears to be very species-specific. Bootstrap values are given for those clusters that contain T-box regulated systems (indicated in black). Those systems that are controlled by a T-box are colored. For the LIVCS-family the sequences of the experimentally studied transporters from Pseudomonas aeruginosa (BraZ [49]), Corynebacterium glutamicum (BrnQ [48]) and Lactobacillus delbrueckii (BrnQ [43]) were included in the analysis. For the APC-family all B. subtilis sequences were included together with the orthologous clusters containing T-box regulated systems. For the MFS- and NSS-family only the orthologous clusters containing T-box regulated systems are shown. In the case of the MFS-family the asterisk indicates that the upstream sequence of these systems contains a box that seems to be degenerated.

A T-box was identified in front of an APC-family protein encoding gene in all the studied *Bacillus *genomes. In *B. subtilis *and *B. licheniformis *the gene *ybvW *is preceded by a Leu T-box, whereas such a box is lacking upstream of the orthologous genes, which are found in *E. faecalis*, *G. kaustophilus *and in *L. lactis *(co-orthologs: *yibG *and *ysjA*) (Figure 3). The Leu T-box suggests that the YbvW protein is a Leucine transporter, in line with the general functionality of transporters of the APC family (family characteristics described in [44,45]). Surprisingly, in the members of the *Bacillus cereus*-group another APC family gene is preceded by a T-box, specific for threonine. Although an orthologous gene is present in most of the Firmicutes genomes, e.g. *ykbA *in *B. subtilis*, it is regulated by a T-box only in the species of the *Bacillus cereus*-group (Figure 3). The protein encoded by *ykbA *in *B. subtilis *has recently been shown to be a Ser/Thr exchanger and was consequently renamed SteT [45]. A similar functionality of the protein ortholog in the members of the *Bacillus cereus *group is supported by the codon identification of the T-box.

#### The LIVCS family

The *Bacilli *of the *Bacillus cereus *group, the *Lactobacilli *and the *Clostridia *contain several branched-chain amino acid cation symporters of the LIVCS-family [46], some of which are T-box regulated (Figure 3). Since the three branched-chain amino acids share very similar molecular properties (e.g. size and hydrophobicity) we expect that these transporters are not highly specific despite their proposed amino acid specific control, but merely that expression of the "multi-specific" system has been brought under the control of the individual amino acids. Indeed, the orthologous transporters that have been characterized in *L. delbrueckii *(BrnQ; [43]), *C. glutamicum *(BrnQ; [47,48]) and *P. aeruginosa *(BraZ; [49]) displayed transport of all three branched-chain amino acids.

#### The NSS family

**Figure 4:**
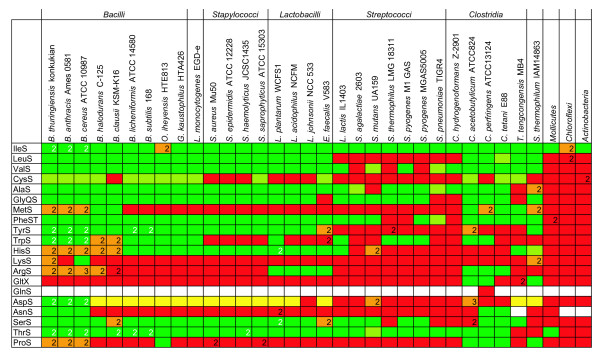
**T-box regulation of tRNA ligase encoding genes in the *Firmicutes*.** The color coding relates to the presence or absence of a T-box upstream of the genes encoding the amino acid-specific tRNA ligases in the various species and strains. Green indicates the tRNA ligase(s) is (are) regulated by a T-box and red that the tRNA ligase(s) is (are) not regulated by a T-box. Although most tRNA ligases are present in one copy on the genome, several organisms contain two, or in some cases three copies of specific ligases (indicated by a number in the box). Orange indicates that 1 of the 2 tRNA ligases is regulated by a T-box or 1 out of 3 in the case of the *argS *genes in *B. cereus *ATCC 10987 and the *aspS *genes in *C. acetobutylicum*. Light green indicates that the tRNA ligase is not the first in the operon, but is regulated by a T-box with the same specificity. Yellow color coding indicates that the regulated tRNA ligase is the second gene in an operon in combination with another tRNA ligase gene regulated by a T-box with different specificity. White indicates that no tRNA ligase of this type is present in the organism. In principle, a species needs at least one specific tRNA ligase for each amino acid. Nevertheless, there are exceptions. For instance, all but one (*Clostridium perfringens*) of the analyzed genomes lack the gene that encodes a Gln-tRNA ligase and the genomes of the *Chloroflexi, Actinobacteria a*nd *Thermoanaerobacter tencongens *also lack an Asn-tRNA ligase. In these cases, the biological role of the Gln-tRNA ligase is taken over by the Glu tRNA ligase, which couples a Glu residue to the tRNA^Gln^. The residue is subsequently transformed into a Gln by a tRNA specific amidotransferase [66]. Similarly, an Asn-tRNA^Asn ^is formed via transamidation of an Asp residue (Asp-tRNA^Asn ^to Asn-tRNA^Asn^) in bacteria that lack an Asn tRNA ligase [67]. Consequently, we found that all species lacking either the Gln-tRNA ligase or the Asn-tRNA ligase have an orthologous gene coding for the corresponding amidotransferase. No T-boxes were identified upstream of those genes.

Finally, the LIVCS family branched-chain amino acid transporter BraZ of *P. aeruginosa*, was shown to have a clear preference for isoleucine and valine over leucine [49]. In this respect it is noteworthy that in the *Bacilli *of the *Bacillus cereus *group the expression of one of the homologs of the NSS family (neurotransmitter:sodium symport) is controlled by a Leu T-box, whereas such a box is lacking for the LIVCS homologs in those species (Figure 4). Besides the Leu T-box regulated NSS transporter, the *Bacilli *of the *Bacillus cereus *group contain three other homologs of the same family, two of which are controlled by a Trp and a Phe T-box, respectively (Figure 3). These two amino acids agree well with the experimentally determined tryptophan transport functionality of the NSS homolog TnaT in *S. thermophilum *[50]. The presence of a regulatory T-box ranging from Leu to Trp, and Phe suggest that the members of the NSS transporter family may display a rather broad amino acid specificity.

#### b) Hypothetical proteins controlled by a T-box

Another class of proteins to which significant functional information could be added (when compared to the NCBI-annotation) using the specificity of the detected T-box is that of the so-called hypothetical proteins or unknown function proteins (data accumulated in Table 2). Obviously, when orthologous proteins in related species were also of unknown function, specifier codon information clearly improved the annotation. Examples of new annotations related to amino acid biosynthesis or transport are enzymes (methionine synthase, cystathionine gamma synthase, chorismate mutase, anthranilate synthase), transporters (Leu-, Lys- and His-specific permeases), tRNA-ligase related functions, and regulation (anti-TRAP protein).

**Table 2:** Proposed annotation of the genes regulated by a T-box that were assigned as "hypothetical protein" in the original NCBI annotation file.

**Species**	**Gene ID**	**T-box**	**Proposed function**
*Bacillus halodurans *C-125	BH0807	Lys	Lysine-specific permease
*Bacillus subtilis *168	BSU02530	Trp	Anti TRAP protein
	BSU34010 (yvbW)	Leu	Leucine-specific permease
*Enterococcus faecalis *V583	EF2480	Gly	Gly related hypothetical
*Lactobacillus acidophilus *NCFM	LBA1071	Ile	Ile related hypothetical
*Lactobacillus johnsonii *NCC 533	LJ0632	Met	5-methyltetrahydropteroyltriglutamate – homocysteine methyltransferase (Methionine synthase)
*Lactobacillus plantarum *WCFS1	lp_3283	Met	5-methyltetrahydropteroyltriglutamate – homocysteine methyltransferase (Methionine synthase)
*Listeria sp*.^1^	lmo1740	His	Histidine transport system permease protein hisM
	lmo2587	Met	Met related cytosolic hypothetical
*Staphylococcus aureus*^2^	SA0347	Met	Cystathionine gamma-synthase
	SA1199	Trp	Anthranilate synthase component I
*Streptococcus agalactiae*^3^	SAG0809	Ala	Ala-tRNA ligase related hypothetical
*Streptococcus pneumoniae*^4^	spr0489	Val	Val-tRNA ligase related hypothetical
	spr1241	Ala	Ala-tRNA ligase related hypothetical
	spr1331	Gly	Gly-tRNA ligase related hypothetical
	spr1471	Thr	Thr-tRNA ligase related hypothetical
	spr1638	Trp	Trp biosynthesis related hypothetical
*Streptococcus thermophilus*^5^	str0474	Val	Val-tRNA ligase related hypothetical
	str1594	Trp	Chorismate mutase

^1 ^*Listeria innocua *Clip11262, *Listeria monocytogenes *EGD-e, *L. monocytogenes *4bF2365. ^2 ^*Staphylococcus aureus *MW2, *S. aureus *N315. ^3 ^*Streptococcus agalactiae *2603, *S. agalactiae *A909, *S. agalactiae *NEM316.^4 ^*Streptococcus pneumoniae *TIGR4, *S. pneumoniae *R6. ^5 ^*Streptococcus thermophilus *CNRZ106, *S. thermophilus *LMG18311.

### III) Taxonomic variation and T-box evolution

The comprehensive list of T-boxes that was generated for all sequenced genomes (see Table 3 for the phylogenetic distribution) confirmed the previous attribution that T-boxes are predominantly encountered in species of the phylum *Firmicutes *(>95% of the hits)[26]. Our analyses uncovered many previously unidentified T-boxes. In species of the class *Mollicutes *two T-box elements were found, but only in the subclass *Endoplasmatales *[51], in *Proteobacteria *(i.e. in *Geobacter sulfurreducens *and *Pelobacter carbinolicus*) a typical T-box element was identified upstream of the *leuA *gene (2-isopropylmalate synthase) in both species. *Deinococcus radiodurans *contained two T-boxes (related to ile and gly t-RNA ligase) whereas species of the phylum of *Chloroflexi *(*Dehalococcoides CBDB1 *and *Dehalococcoides ethenogenes *195) contained three T-box elements (one related to an ile tRNA ligase and two related to tryptophan biosynthesis (*trpE *and *trpB*-like). Earlier analysis of riboswitches in Actinobacteria showed that some species belonging to this phylum contain a T-box upstream of *ileS *[52]. However, *Symbiobacterium thermophilum *contained not less than eighteen T-boxes, comparable to species of the *Firmicutes*. This finding is in line with the conclusion of [53] that *S. thermophilum *is probably more closely related to *Firmicutes *than to *Actinobacteria*.

**Table 3:** The occurrence of T-boxes in different bacterial phyla. The phyla are taken from the NCBI taxonomy [68].

Phylum	Genomes sequenced	Genomes with at least one T-box	Number of T-boxes
Firmicutes	53	53	855
Actinobacteria	19	12	32
Chloroflexi	2	2	6
Deinococcus/Thermus	3	1	2
Proteobacteria	125	2	2
Cyanobacteria	13	0	0
Chlamydiae	10	0	0
Bacteroidetes/Chlorobi	7	0	0
Spirochaetes	6	0	0
Planctomycetes	1	0	0
Aquificiates	1	0	0
Fusobacteria/Thermotogae	2	0	0

Total	242	70	897

To evaluate the phylogenetic distribution of T-boxes in more detail, the correlation between the presence of T-box regulatory elements and the regulated genes was analyzed for the Firmicutes and will be described shortly in the next sections. Furthermore, the scattered appearance of these regulatory boxes as observed for the various transporter families will be discussed.

#### T-box regulation of genes encoding tRNA ligases in the Firmicutes

It appeared (Figure 4) that regulation by T-boxes is conserved in almost all (at least 29 out of 34) *Firmicutes *for several tRNA ligases (*ileS*, a *laS*, *serS *and *thrS*), whereas some tRNA ligases (*lysS, asnS *and *gltX*; the latter gene encodes a Glu-tRNA ligase (see also the legend of Figure 4)) appeared to be controlled by a T-box in only a few species. The genes encoding the tRNA ligases for cysteine and asparagine were often found as the second gene in a putative operon that was T-box regulated. In several organisms, multiple copies of amino-acid specific tRNA-ligase encoding genes are found and in more than half of the cases (58%) only one of them is subject to T-box regulation.

A clear phylogenetic effect is observed when the four major orders within the *Firmicutes *(*Bacillales*, *Clostridia*, *Lactobacillales *and *Mollicutes*) are compared. This is true for both the number and the type of tRNA-ligase encoding genes regulated by a T-box. Most T-box regulated tRNA-ligase encoding genes are found in the *Bacillales *and especially in species of the *Bacillus cereus *group. Within the *Lactobacillales*, it appears that *Streptococci *have far less tRNA- ligase encoding genes regulated by T-boxes than *Lactobacillus *species. The lowest number of tRNA ligases regulated by T-boxes was found for *S. thermophilus, S. pneumoniae *and some strains of *S. pyogenes*. The relatively low amount of T-box regulation in these species could be the result of regressive evolution, a process that was suggested to be the underlying mechanism for the large loss of functionally active genes in *S. thermophilus *[54].

#### T-box regulation of genes involved in amino acid biosynthesis in the Firmicutes

**Figure 5:**
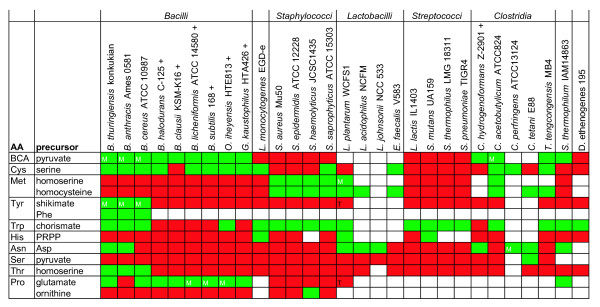
**T-boxes preceding the genes related to amino acid biosynthesis in *Firmicutes*.** Color coding identifies the presence of the biosynthesis pathway and whether it is regulated by a T-box: Green; T-box regulated; red; not T-box regulated; no color; pathway absent. ^+^TRAP protein is present. ^M ^Pathway genes organized in multiple operons. BCA indicates the branched chain amino acids valine, leucine and isoleucine.

T-box regulation of genes related to amino acid biosynthesis has been described previously for various amino acids [3,23,24]. Like in the case of the t-RNA ligases, T-box control of amino acid biosynthesis displayed clear phylogenetic patterns (Figure 5). For instance, the biosynthesis of Branched Chain Amino Acids (BCA: isoleucine, leucine, valine) was found to be T-box regulated in *Bacillales *and *Clostridia*, whereas several families within the *Bacillales *(e.g. *Staphylococci *and *Listeria*) as well as several *Streptococci *consistently lack T-box control of BCA biosynthesis. Similarly, we found that the species of the *B. cereus *group contain a T-box in the upstream region of the tyrosine biosynthesis operon consistent with the experimental data that showed that tyrosine biosynthesis from shikimate is T-box regulated in *B. anthracis *[24]. The *B. cereus *group representatives are the only organisms in our study that encode a phenylalanine-4-hydroxylase ortholog (converting phenylalanine into tyrosine). We found this gene also to be T-box regulated in all members of the *B. cereus *group.

#### The evolution and propagation of T-boxes

An important observation related to T-box evolution was made by Grundy et al. [9,12]. They showed that a single nucleotide change in the specifier codon of the Tyr T-box of *tyrS *in *B. subtilis *was enough to change the amino acid specificity. In addition, while analyzing the distribution of T-boxes over the various transporter families we were struck by the fact that although some of these families are very large, there was only one (or a few) family-member(s) found to be regulated by a T-box and only in a restricted number of species (see Table 1 and e.g. Figure 3). In our opinion, the only likely scenario to explain the phylogenetically limited occurrence of the transporter T-box associations that would not imply massive loss of the T-box regulation was that of acquisition of the regulatory element by the transporter encoding gene in a specific lineage. Moreover, the results of Grundy et al. [9,12] imply that in principle the T-Box can change specificity easily.

**Figure 6:**
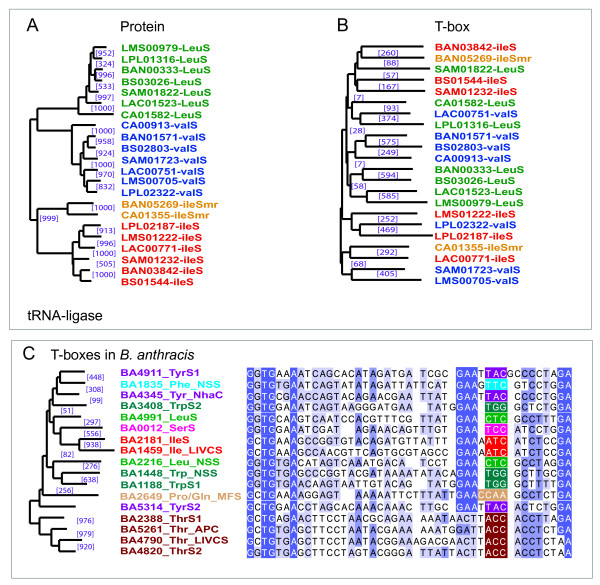
**The evolutionary relationship between some T-boxes.** (A) shows a putative phylogeny of the branched-chain amino acid tRNA ligases of *B. anthracis *Ames, *B. subtilis *168, *C. acetobutylicum *ATCC824D, *L. acidophilus *NCFM, *L. plantarum *WCFS1, *L. mesenteroides *ATCC8293 and *S. aureus *Mu50. (B) shows the Neighbor-Joining tree for the related T-boxes. The underlying alignments were made with the complete 200 – 300 nucleotides of the identified T-boxes. These alignments were homogeneous in the sense that the fully conserved motifs aligned perfectly and that between those conserved elements there were little gaps. Nevertheless, the low bootstrap support for the various branches indicates that this tree is unlikely to reflect the true phylogeny of the regulatory elements. (C) shows the Neighbor-Joining tree for various T-boxes found in *B. anthracis *Ames. Next to the tree, the part of the corresponding multiple sequence alignment containing the specifier codon (indicated in white letters) is depicted. The amino acid specificity of the specifier codon is color-coded: Red and orange relate to Ile, green to Leu, light blue to Phe, beige to Pro or Gln, pink to Ser, brown to Thr, turquoise to Trp, purple to Tyr and dark blue to Val. The family of the protein encoded by the regulated gene is indicated by the letters that follow the amino acid code. These protein families included the APC, LIVCS, MFS, NhaC and NSS transporter protein families and various tRNA-ligase families (S or Smr for mupirocin resistant tRNA ligase). The NSS-family transport proteins regulated by a Leu, Phe and Trp T-box are in-paralogs characteristic for the species of the *Bacillus cereus *group. The purple numbers between brackets indicate the bootstrap support for the displayed clusters (out of 1000).

To analyze this further, the T-boxes preceding the genes that encode the t-RNA-ligases for the branched-chain amino acids (*ileS*, *leuS *and *valS*) were examined. These were chosen because T-box regulation of these genes is most wide-spread (Figure 4) and because the proximal genes themselves, *ileS*, *leuS *and *valS *form a separate tRNA-ligase sub-family with a very clear evolutionary lineage (Figure 6A). In sharp contrast, the NJ-tree of the aligned complete T-boxes (approximately 200 – 300 nt) appeared extremely unreliable (Figure 6B). This finding implies that amino acid specificity of a T-box is not prominent on the overall sequence level and that the apparent overall sequence variability in time of the T-box is relatively high. Simultaneously, in case T-box sequences do cluster in reliable clusters (high bootstrap support) in a NJ-tree they must therefore be closely related in time.

To limit possible obscuring effects of comparing sequences between species, we collected and compared the T-box sequences within species and for clarity restricted the comparison to the T-boxes that accompany transport systems and the related tRNA ligases. For all three analyzed species: *B. anthracis, L. acidophilus *and *L. plantarum*. similar phenomena were observed. The multiple sequence alignment of the analyzed T-box sequences and the associated NJ-tree (depicted in Figure 6C for *B. anthracis*) are strongly suggestive of a close evolutionary relationship between several of the T-boxes. For example in *B. anthracis*, the Thr T-boxes found in front of *BA4970 *(transport systems of LIVCS-type) and the Thr-tRNA ligase (*BA4820*) were highly similar and the same was observed for the Ile T-boxes found in front of another LIVCS homolog and the Ile-tRNA ligase. As the LIVCS homologs appear closely related in time (apparent duplication in the *B. cereus *group ancestor, see Figure 3), the data imply that the regulatory T-box was not inherited in a similar way but 'acquired' independently. In fact, this explanation fits the observed scattered appearance of the T-boxes for the various transporter families perfectly.

The results presented in Figure 6 are also suggestive of another way in which the T-boxes have evolved. The NJ-tree relates the Phe T-box found in front of one of the NSS family transporters to the Tyr T-box associated with the Tyr-tRNA ligase (Figure 6C). It thus seems that the Tyr T-box of the tRNA ligase was duplicated -as this T-box is present in various Firmicutes species- and has diverged/adapted to control a Phe transporter in the Bacilli of the *B. cereus *group. In fact, the similarity between the T-box upstream of Tyr-tRNA ligase and the Phe T-box in front of the transporter is higher than between the Tyr T-box and the Tyr T-box preceding *BA4353 *(NhaC family transporter). This is consistent with the fact that: the Tyr T-box acquisition of the NhaC ortholog should have occurred earlier in history, as the Tyr T-box control of the NhaC ortholog is present in several *Firmicutes*, and the sequences thus had more time to diverge.

## Conclusion

The sequence signature of a T-box is very specific and as a result T-boxes can be readily identified. Using specific T-box HMMs, we identified a large number of the T-boxes and their amino acid specificity in sequenced prokaryote genomes.

An important aspect of this work is that we show that the prediction of the amino acid specificity of the various T-boxes can be used to improve the functional annotation of a large number of genes. In particular, the functional annotation of genes related to amino acid transport and genes with unknown substrate specificity, genes for which it is normally quite difficult to find functional attributes, could be improved significantly. In our opinion, the procedure of improving annotation through knowledge of the regulatory signals can be generalized and should be used on a much broader scale than currently is being done.

Riboswitches have been argued to be among the oldest regulatory systems in bacteria because of their independence of regulatory proteins and widespread biological distribution [35]. One might therefore have expected that T-boxes are abundantly present among all different lineages of bacteria. This clearly can not be concluded from our results and those presented in other studies [52,55]. In fact, these regulatory elements can only be found in a few bacterial phyla and only abundantly in the phylum *Firmicutes*. This implies that either *Firmicutes *developed T-box regulation after their branching off from the other bacteria or that the other bacteria lost the system soon after the branching off of the *Firmicutes *to evolve more complex regulatory systems. Which of the two scenarios is most likely remains unclear.

Nevertheless our data do allow some extrapolation of the propagation of T-boxes within the phylum Firmicutes. We conclude on basis of our observations that the T-boxes have evolved in four clearly distinct ways: i) by co-evolution with the regulated gene or operon; ii) by co-evolution and divergence with the regulated gene or operon to adopt a new specificity; by iii) duplication and insertion of the regulatory element in front of a gene or operon that encodes functions related to the T-box-specified amino acid; and finally iv) by duplication and divergence toward a new amino acid specificity after duplication. In short, this means that every T-box regulatory element acts as a connected yet independent "functional module". The fact that the isoleucine specific T-box connected to the ile-tRNA ligase encoding gene is the only box present in all T-box containing bacteria suggests this box could very well compose the archetype T-box.

## Methods

### Sequence information and tools

Genome sequences and annotation files of completely sequenced bacterial and archaeal genomes were downloaded from the NCBI repository [56]. In addition, for the analysis of the molecular functions encoded by T-box controlled genes, genomic information was obtained from the ERGO genome analysis and discovery system [57]. Multiple sequence alignments were created with MUSCLE [58] and bootstrapped Neighbor-Joining trees with CLUSTALX [59] (corrected for multiple substitutions). HMMs were constructed from a multiple sequence alignment using HMMER 2.3.2 [60]. Conserved nucleotide sequence motifs were recovered via MEME [61] (settings: maximally 5 motifs, modus ZOOPs, minimal width 10 nt, maximal width 30 nt) and visualized using Weblogo [62]. Hidden Markov Models (HMMs) were constructed on basis of the recovered motifs to perform genome-wide searches. Although HMMsearch [60] yielded essentially identical results to the search-tool MAST [63], in our hands the procedure was much faster and the output was easier to interpret (including motif e-values rather than p-values). RNA secondary structure predictions were performed by Mfold [38] using default settings.

### Identification of a general T-box sequence

The T-boxes reported so far in literature were mainly located upstream of tRNA-ligase encoding genes in species of the phylum *Firmicutes *[7,26]. We therefore initiated the search for a general T-box sequence motif by collecting the nucleotide sequences (300 nt) preceding all tRNA ligases (n = 910) found in sequenced genomes of the *Firmicutes*. Five characteristic consecutive motifs were recovered in the nucleotide sequences (displayed in Figure 1). They were part of a generic T-box sequence that spans a length of about 250 nt, as described by [8]. The best-conserved motif (Figure 1, motif 1, E-value = 1.2e^-2841^), which is located closest to the translation start, had a length of 30 nt and was found in about 61% (n = 553) of the nucleotide sequences. A genome-wide HMMsearch based upon the best-conserved motif yielded 374 new hits. To probe eventual in-homogeneity induced by the fact that only T-boxes associated with tRNA ligases were taken to create the initial T-box HMM, a new HMM based on the non-tRNA ligase associated T-boxes was made and the genomes were searched anew. We found that all t-RNA ligase associated T-boxes were recovered (with an e-value < 1) with this new HMM. This finding implied that the set of recovered T-boxes was relatively homogeneous. The upstream 500 nt of every putative best-conserved T-box motif was then checked for the presence of at least one of the four other conserved T-box specific motifs recovered using MEME. In 40 cases (less than 4% of total) none of the other motifs was detected. Without exception, these were not located in the proximity of a coding sequence and thus unlikely to be related to transcription attenuation. The hits were therefore considered false-positives and were removed. To potentially increase the recovery rate, the remaining 887 T-box sequences were subdivided on basis of the taxonomy of the species and taxonomic class-specific HMMs were built as before. This procedure yielded only 10 additional T-boxes. Finally, the position of all boxes with respect to the start-codon of the proximal gene was determined. It appeared that In 839 cases, motif 1 was found to be located within 300 nucleotides upstream of a predicted gene start. The remaining 48 T-boxes were located 300 to 480 nt upstream of a start-codon. Manual inspection of those T-boxes revealed an overrepresentation of the proximal genes *thrZ *(11×), *trpE *(11×), branched amino acid transferase (5×), chorismate mutase (5×) and a sodium symporter (4×). In fact, for both *thrZ *and *trpE *it was previously shown that they are preceeded by multiple (2 or 3) adjacently located T-boxes in different *bacilli *[3,29].

### Functional classification of the genes regulated by T-boxes

The genes downstream of the recovered T-boxes were divided into four different classes on basis of the gene annotation information of the proximal gene: 'tRNA ligation', 'amino acid biosynthesis, 'amino acid transport' and 'other'. In those cases the T-box preceded an operon (45%) it appeared that most of these operons (>75%) contained genes of only one functional class.

### Comparison with the Rfam T-box model

The Rfam database [36] is an excellent reference database to start the identification of specific RNA-motifs. To evaluate the performance of our recovery procedure, we compared our results with predictions based on the Rfam T-box HMM. Unfortunately, a direct comparison with the contents of the database proved not informative as the Rfam database includes all nucleotide sequences present in the TREMBL database, which includes many genome fragments or partially sequenced genomes, whereas our analysis was restricted to completed genomes.

Therefore, we used the Rfam model to search for T-boxes against the same set of complete genome sequences that was used for our analysis. A striking difference between the Rfam-search and our analysis was that the Rfam-search was computationally far more expensive (more than three weeks on an 8 node,16 core, linux cluster compared to 16 hours on a 2 core linux system). For the selected species we predicted 883 T-boxes characterized by the presence of all the characteristic motifs, of which 835 (95%) were within the first 300 nt upstream of a gene start. When using a cut-off of 53.000 bits (described by Rfam as reliable) only 501 (60%) of the 883 T-boxes were predicted using Rfam. In addition to these 501 shared T-boxes Rfam identified 8 additional genuine T-boxes (~0.9 gain) within the first 300 nt. upstream of a gene start. At a lower cutoff-value (25.000 bits), chosen such that ~81% (683) of the boxes identified by us were recovered, the prediction with our HMM and the Rfam results were more alike albeit that Rfam now also yielded a considerable number of false positive identifications: only ~90% of the total number of T-boxes was found within the first 300 nt. upstream of a gene start. Using this cutoff, 6 additional genuine T-boxes were found (~0.7% gain) to be present within the first 300 nt. upstream of a gene start. When applying no cutoff, the Rfam-search retrieved 89% (745) of the T-boxes that were found in our analysis. However, in this case the number of putative false-positives (not located within 300 nt upstream of a gene start) had increased to 65% of the total.

## Authors' contributions

MW conceived, designed and carried out the tRNA-ligase and amino acid biosynthesis analysis, Rfam comparison and drafted and revised the manuscript. TGK carried out the T-box detection and specificity analysis, RJS and MK helped in the design and coordination of the research and drafting and revising the manuscript. CF helped in the design and coordination of the research, carried out the transporter analysis and helped drafting and revising the manuscript. All authors have read and approved the final manuscript

## Supplementary Material

Additional file 1 T-box location organized per genome. The occurrence of T-boxes in all analyzed genomes is shown in detail. Position, direction, e-value and specifier codon are shown for the T-box, together with position, name and function of the first gene located downstream and the size of the operon located downstream. All T-boxes are color coded according to the function of the genes located downstream: red; tRNA synthesis; green; amino acid biosynthesis; blue; amino acid transport and purple: other/unknown. Click here for file

Additional file 2 T-box distribution among different bacterial species. For all species the number of T-boxes is displayed, divided over four different categories (tRNA synthesis, amino acid transport, amino acid biosynthesis and other). Between brackets the number of regulated genes is shown. This number is based on the operon structure of the genes downstream of the T-box. In case multiple strains of a specific species were sequenced, these are only shown when differences between the strains were observed. Click here for file

Additional file 3 T-box enhanced functional annotation of amino acid transport. Extensive description of the improved annotation of the T-box regulated transporter systems not given in the main text. Click here for file
