# Supplementary material for: An in silico analysis of T-box regulated genes and T-box evolution in prokaryotes, with emphasis on prediction of substrate specificity of transporters
Source: BMC Genomics. 2008 Jul 14;9:330. doi: 10.1186/1471-2164-9-330 (PMC2494555; doi:10.1186/1471-2164-9-330)
Supplement: Additional file 3 — T-box enhanced functional annotation of amino acid transport. Extensive description of the improved annotation of the T-box regulated transporter systems not given in the main text. [file 1471-2164-9-330-S3.pdf]

Additional file 3: *T-box enhanced functional annotation of amino acid transport*.

Many of the genes encoding amino acid transporters were found to be preceded by a T-box, especially in the genomes of the *Lactobacilli* and *Bacilli* of the *Bacillus cereus*-group. The transporters controlled by T-boxes belonged to no less than seven distinct transporter families (MFS, 2.A.1; APC, 2.A.3; NSS, 2.A.22; DAACS, 2.A.23; LIVCS, 2.A.26; NhaC, 2.A.35; and ABC-cassette, 3.A.1). These families are related to the two main transporter sub-classes “porters” and “P-P bond hydrolysis driven”, according to the Transporter Classification described by Saier [1]. The first class of transport systems uses membrane potential (via cation symport and solute antiport) to acquire solutes, whereas the second class of transport systems apply the free-energy obtained by the hydrolysis of ATP.

The ABC family T-box regulation of ABC transport systems was found in most lineages of the *Firmicutes* but not in the *Bacilli.* The T-box regulated ABC amino acid transporters could be sub-divided into four sub-families, based on the specificity of the substrate-binding protein and the permease: the HAAT sub-family (3.A.1.4; hydrophobic amino acids), the PAAT sub-family (3.A.1.3; polar amino acids), the PEPT sub-family (3.A.1.5; peptides) and the MUT sub-family (3.A.1.24; methionine). Representatives of the latter three sub-families are present in the genome of *Lactobacillus plantarum* WCFS1*.* In total, the genome of *L. plantarum* contains five members of the PAAT sub-family, which were

originally annotated as glutamine transport systems [2] based on the relatively high sequence similarity with the experimentally verified glutamine transporter of *E. coli* (GlnHPQ; [3, 4]). However, one of the corresponding operons (genes lp_2312 - lp_2314) is preceded by a His T-box, strongly suggestive of a biological role in the transport of histidine instead. The *hisJ* orthologs (HisJ is the histidine-binding protein characterized in *E. coli* [5]) from *E. faecalis*, *L. acidophilus* and *L monocytogenes* are also controlled by a His T-box. Therefore, we propose to change the functional annotation of the corresponding system in *L. plantarum* to ABC histidine transport system.

Another striking connection for T-box regulated ABC transporters in *L. plantarum* was observed with methionine metabolism. Four ABC systems were found linked to this amino acid. One of the PAAT sub-family systems, encoded by the operon *lp_3209 – lp_3211*, is orthologous to the YckKJL cystine uptake system in *B. subtilis* [6]. Although the operon itself is not regulated by a T-box, it is followed directly on the genome by gene *lp_3214*, which is regulated by a methionine T-box. Phylogenetic analysis suggested that genes *lp_3209* and *lp_3214* are in-paralogs, and therefore should encode very similar molecular functions. Furthermore, the Met T-box indicated that the substrate of Lp_3214 should be related to methionine uptake and/or synthesis. Inspection of the KEGG map displaying methionine metabolism (see map 00271, [7] or Fig 2) yielded a good candidate molecule, namely ‘cysthationine’. This molecule is an important precursor in the biosynthesis of methionine and is structurally very similar to

cystine. One could therefore speculate that Lp_3214 is a cysthationine-binding protein. Inspection of the protein sequence revealed another possible explanation for the presence of the two in-paralogs. Whereas the cystine-binding protein Lp_3209 has five methionine residues in its sequence, the T-box regulated in-paralogous protein Lp_3214 has only a starting methionine. The other four methionine residues have been replaced by isoleucine or leucine. Thus, the molecular function of the two in-paralogs could be identical but their biological role could differ in the sense that in the near absence of methionine, as signaled by uncharged t-RNA, a cystine-binding protein could still be produced.

Another example of Met T-box regulation in *L. plantarum* is the two systems of the MUT sub-family which are both preceded by a Met T-box (*lp_1744 – lp_1746* and *lp_2350 – lp_2352*). The system encoded by the operon *lp_1744 – lp_1746* is orthologous to the experimentally characterized system encoded by *metNPQ* of *B. subtilis* [8] and as such is expected to transport methionine sulfoxide, D- and L-methionine. Considering the similarity in regulation (i.e. the inducing signal) we expect the paralogous system to have slightly altered substrate specificity. The fourth ABC system (*lp_0148 – lp_0150*) regulated by a Met T-box in *L. plantarum* is not involved in the transport of methionine but merely in the transport of cobalt or cobalamine, which is a co-factor for the conversion of homocysteine into methionine by MetH (reaction EC 2.1.1.13; [9])(Fig. 2). In concordance, we found that the operon containing the *metH* gene (*lp_1374*) is also controlled by a Met T-box. It thus appears that in the absence of methionine,

L. plantarum uses a single mechanism to switch on not only a transport system for the amino acid itself, but also for the precursors and co-factors needed in its biosynthesis (see Fig 2).

The NhaC family In contrast to the APC family transporters, the NhaC family is rather small and Firmicutes contain only one (*e.g. Clostridium acetobutylicum*, *Staphylococci*), two (*e.g. C. difficile*, *E. faecalis*, several *Bacilli* and *Lactobacilli*) or three (e.g. *B. cereus*-group) homologs [10]. In most of these species one of the homologous genes is controlled by a Tyr T-box, and in *L. plantarum* both paralogous genes. In contrast, the homologous genes in Staphylococci and in *B. subtilis*, *B. licheniformis* and *B. clausii* lack a T-box. The presence of a Tyr T-box suggests the related proteins play a role in the import, or eventually the production, of tyrosine. Surprisingly, the NhaC family homologs present in *B. firmus* (gene *nhaC*) [11, 12] and *B. subtilis* (genes *yheL* and *yqkI*) [13] have been identified as Na+/H+ antiporter (NhaC/YheL) or malic acid/sodium lactate antiporter (YqkI), respectively. It was not directly clear how the latter functionalities should be connected to the uptake or synthesis of tyrosine. The T-box regulated NhaC family homologs are most similar to the *B. firmus* NhaC and thus are expected to display similar/identical molecular function (i.e. Na+/H+ antiport). However, considering the fact that the malic acid/sodium lactate antiporter of the same family has Na+/H+ antiport functionality [13] and that NhaC of *B. firmus* was not extensively characterized for substrate specificity [11, 12] it could very well be that the latter transporter operates as an acid/ salt

antiporter, where the acid is very similar to malic acid in structure and the salt to sodium lactate. Given the fact that one of the final steps in the biosynthesis of tyrosine is a transamination reaction with glutamate or aspartate as source of the amino-group and that aspartic acid is structurally very similar to malic acid, this hypothesis is appealing. While such a functional annotation remains speculative, the presence of the T box does imply a biological role in amino acid biosynthesis instead of Na^+/H^+ homeostasis as was previously suggested [13].

References

1. Saier MH, Jr.: **A functional-phylogenetic classification system for transmembrane solute transporters**. *Microbiol Mol Biol Rev* 2000, **64**(2):354-411.
2. Kleerebezem M, Boekhorst J, van Kranenburg R, Molenaar D, Kuipers OP, Leer R, Tarchini R, Peters SA, Sandbrink HM, Fiers MW *et al*: **Complete genome sequence of Lactobacillus plantarum WCFS1**. *Proc Natl Acad Sci U S A* 2003, **100**(4):1990-1995.
3. Weiner JH, Furlong CE, Heppel LA: **A binding protein for L-glutamine and its relation to active transport in E. coli**. *Arch Biochem Biophys* 1971, **142**(2):715-717.
4. Hsiao CD, Sun YJ, Rose J, Wang BC: **The crystal structure of glutamine-binding protein from Escherichia coli**. *J Mol Biol* 1996, **262**(2):225-242.
5. Oh BH, Kang CH, De Bondt H, Kim SH, Nikaido K, Joshi AK, Ames GF: **The bacterial periplasmic histidine-binding protein. structure/function analysis of the ligand-binding site and comparison with related proteins**. *J Biol Chem* 1994, **269**(6):4135-4143.
6. Burguiere P, Auger S, Hullo MF, Danchin A, Martin-Verstraete I: **Three different systems participate in L-cystine uptake in Bacillus subtilis**. *J Bacteriol* 2004, **186**(15):4875-4884.
7. Kanehisa M, Goto S, Hattori M, Aoki-Kinoshita KF, Itoh M, Kawashima S, Katayama T, Araki M, Hirakawa M: **From genomics to chemical genomics: new developments in KEGG**. *Nucleic Acids Res* 2006, **34**(Database issue):D354-357.
8. Hullo MF, Auger S, Dassa E, Danchin A, Martin-Verstraete I: **The metNPQ operon of Bacillus subtilis encodes an ABC permease transporting methionine sulfoxide, D- and L-methionine**. *Res Microbiol* 2004, **155**(2):80-86.
9. Drummond JT, Huang S, Blumenthal RM, Matthews RG: **Assignment of enzymatic function to specific protein regions of cobalamin-dependent methionine synthase from Escherichia coli**. *Biochemistry* 1993, **32**(36):9290-9295.
10. Ren Q, Kang KH, Paulsen IT: **TransportDB: a relational database of cellular membrane transport systems**. *Nucleic Acids Res* 2004, **32**(Database issue):D284-288.
11. Ivey DM, Guffanti AA, Bossewitch JS, Padan E, Krulwich TA: **Molecular cloning and sequencing of a gene from alkaliphilic Bacillus firmus OF4 that functionally complements an Escherichia coli strain carrying a deletion in the nhaA Na+/H+ antiporter gene**. *J Biol Chem* 1991, **266**(34):23483-23489.
12. Ito M, Guffanti AA, Zemsky J, Ivey DM, Krulwich TA: **Role of the nhaC-encoded Na+/H+ antiporter of alkaliphilic Bacillus firmus OF4**. *J Bacteriol* 1997, **179**(12):3851-3857.

13. Wei Y, Guffanti AA, Ito M, Krulwich TA: **Bacillus subtilis YqkI is a novel malic/Na+-lactate antiporter that enhances growth on malate at low protonmotive force**. *J Biol Chem* 2000, **275**(39):30287-30292.
